# Reactive Neuroblastosis in Huntington’s Disease: A Putative Therapeutic Target for Striatal Regeneration in the Adult Brain

**DOI:** 10.3389/fncel.2018.00037

**Published:** 2018-03-09

**Authors:** Mahesh Kandasamy, Ludwig Aigner

**Affiliations:** ^1^Laboratory of Stem Cells and Neuroregeneration, Department of Animal Science, School of Life Sciences, Bharathidasan University, Tiruchirappalli, India; ^2^Faculty Recharge Programme, University Grants Commission (UGC-FRP), New Delhi, India; ^3^Institute of Molecular Regenerative Medicine, Paracelsus Medical University, Salzburg, Austria; ^4^Spinal Cord Injury and Tissue Regeneration Center, Paracelsus Medical University, Salzburg, Austria

**Keywords:** Huntington’s disease, adult neurogenesis, striatum, reactive neuroblastosis, doublecortin

## Abstract

The cellular and molecular mechanisms underlying the reciprocal relationship between adult neurogenesis, cognitive and motor functions have been an important focus of investigation in the establishment of effective neural replacement therapies for neurodegenerative disorders. While neuronal loss, reactive gliosis and defects in the self-repair capacity have extensively been characterized in neurodegenerative disorders, the transient excess production of neuroblasts detected in the adult striatum of animal models of Huntington’s disease (HD) and in post-mortem brain of HD patients, has only marginally been addressed. This abnormal cellular response in the striatum appears to originate from the selective proliferation and ectopic migration of neuroblasts derived from the subventricular zone (SVZ). Based on and in line with the term “reactive astrogliosis”, we propose to name the observed cellular event “reactive neuroblastosis”. Although, the functional relevance of reactive neuroblastosis is unknown, we speculate that this process may provide support for the tissue regeneration in compensating the structural and physiological functions of the striatum in lieu of aging or of the neurodegenerative process. Thus, in this review article, we comprehend different possibilities for the regulation of striatal neurogenesis, neuroblastosis and their functional relevance in the context of HD.

## Introduction

Huntington’s disease (HD) is an adult onset, progressive neurodegenerative syndrome that has clinically been characterized by chorea, dementia and psychiatric illness (Walker, 2007). Historically, symptoms of chorea had been observed by many physicians (Lanska, 2010), while George Huntington portrayed the clinical symptoms and provided the evidence for the hereditary nature of HD in 1872 (Huntington, 1872; Lanska, 2000). Since then, an enormous scientific progress has been made in understanding the biochemical, molecular genetics and pathological basis of HD worldwide (Wexler et al., 2004; Bates, 2005; Moily et al., 2014). In 1983, the HD Collaborative Research Group, under the direction of Nancy Wexler, successfully mapped the defective gene responsible for HD to chromosome 4p16.3 (Gusella et al., 1983). In 1993, the disease pathogenic mutation has been recognized as a polymorphic CAG-repeat expansion in the exon 1 of the HD gene (The Huntington’s Disease Collaborative Research Group, 1993). The physiological role of the wild-type (WT) HD gene remains unclear. However, several lines of experimental evidence of gene knockout paradigms suggested that the expression of WT HD gene is indispensable for embryogenesis, vesicular trafficking, synaptic plasticity and neuroprotection (Duyao et al., 1995; Dragatsis et al., 2000; Reiner et al., 2003). The unstable CAG repeat expansion of more than 35–39 in the HD gene is translated into polyglutamine (polyQ) stretches in the huntingtin protein (Bates, 2003; Cornett et al., 2005; Moily et al., 2014). The abnormal polyQ repeat sequence is known to cause misfolding and aggregation of the huntingtin protein (DiFiglia et al., 1997; Bates, 2005; Poirier et al., 2005) leading to the selective degeneration of medium spiny neurons (MSNs) in the striatum and onset of the disease (Graveland et al., 1985). Consequently, neurotransmitter dysfunction, oxidative stress, microglial activation, reactive astrogliosis have been characterized as second degree of pathological consequences in the striatum of HD subjects (Walker, 2007; Velusamy et al., 2017; McColgan and Tabrizi, 2018). It has been predicted that recent advancements in CRISPR/Cas9 genome-editing tools and patient-specific generation of induced pluripotent stem cells (iPSCs) might significantly contribute to the development of future gene therapies for HD (Xu et al., 2017). Nevertheless, refining mechanisms of the existing self-regenerative process of the adult brain, namely adult neurogenesis, holds great promise for the establishment of non-invasive clinical procedures to treat HD.

## Migration of Neuroblasts in the Healthy Adult Forebrain

The subventricular zone (SVZ) is a prime neuropoietic niche of the brain responsible for the postnatal neurogenesis in the telencephalon (Doetsch et al., 1997, 1999). In the adulthood, the SVZ continues to harbor a heterogeneous population of neural stem cells (NSCs) that generates polarized neuroblast progenies, migrating through the rostral migratory stream (RMS) into the olfactory bulb (OB), where they terminally mature into functional interneurons (Doetsch et al., 1997, 1999; Gritti et al., 2002; Ming and Song, 2011). While neurogenesis in the human hippocampus has generally been recognized and accepted (Eriksson et al., 1998), the incidence of olfactory neurogenesis in the human brain has been an ongoing subject of debate (Kirschenbaum et al., 1999; Pagano et al., 2000; Curtis et al., 2007; Sanai et al., 2011; Ernst et al., 2014). A recent report by the Mechawar group provided evidence for the occurrence of doublecortin (DCX) positive neuroblasts in the SVZ-OB path in the post-mortem brains from suicide subjects (Maheu et al., 2015). However, the evidence for the migration of neuroblasts and mechanisms underlying their migration towards the OB in the normal human brain are yet to be validated. In non-primate mammalian brains, the glial tube structure of the RMS provides a scaffold platform for the migrating neuroblasts towards the OB (Lois and Alvarez-Buylla, 1994; Doetsch et al., 1999; Ming and Song, 2011). A reciprocal interaction between the neuroblasts and glial cells through the assistance of cell surface adhesion molecules, extracellular matrix, metalloproteases, transcription factors, neurotransmitters, neurotrophins and chemo-attractants have been suggested to mediate this distinct long-distance cell migratory process in the adult forebrain (Gritti et al., 2002; Ghashghaei et al., 2007; Ming and Song, 2011). Besides the glial tube, directional flow of the cerebrospinal fluid (CSF) mediated by the ciliary movement of ependymal cells in the ventricle has been proposed to play a critical role in the migration of neuroblasts along the SVZ—RMS-OB path (Sawamoto et al., 2006). For yet unknown reasons, the RMS structure in the human brain seems to be restricted to the developmental stage and absent in the adulthood (Kam et al., 2009; Sanai et al., 2011; Wang et al., 2011).

## Neurogenesis and Neuroblastosis in the Adult HD Striatum

**Figure 1:**
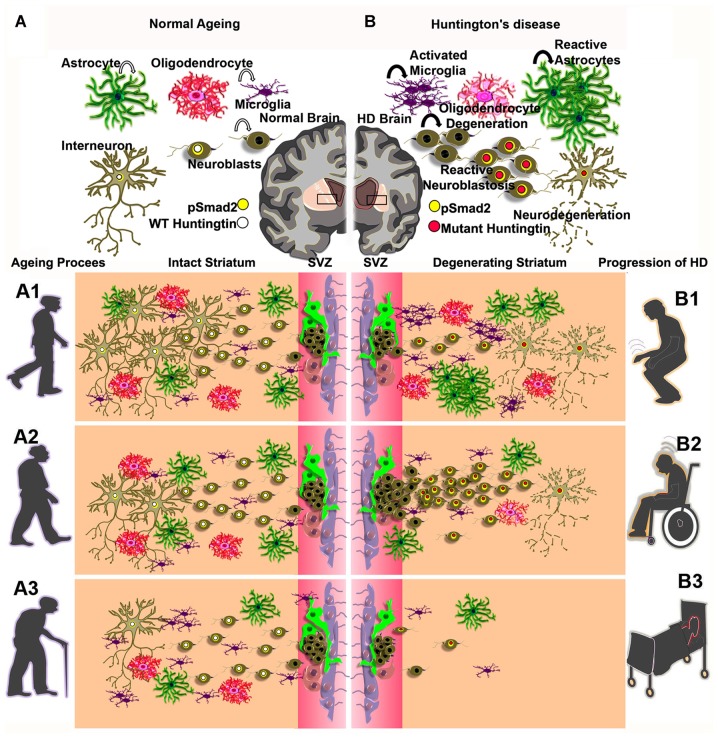
Graphical illustration of cell populations of the CNS—astrocyte (dark green), oligodendrocyte (pinkish red), neuroblasts (drab), interneuron (olive) and microglia (indigo) in the adult brain during normal aging process **(A)** and activated microglia, reactive astrocytes and reactive neuroblastosis and neurodegeneration in the degenerating striatum of Huntington’s disease (HD; **B**). Concentric circles of yellow and white indicate a possible overlap between pSmad2 (Yellow) and wild-type (WT) huntingtin protein (white; **A**) or mutant huntingtin protein (Red; **B**). **(A1–A3)** represent a gradual decline of neurogensis in the subventricular zone (SVZ)-striatal regions upon aging process. **(B1–B3)** illustrate abnormal neurogenic events and neuro degeneration in the SVZ-striatal regions in early onset, mid and late stages of HD.

Physical exercise and environmental enrichment paradigms have been shown to positively influence hippocampal neurogenesis in the healthy brain (Kempermann et al., 1997; van Praag et al., 1999). While the SVZ of the adult brain is highly refractory to external stimuli in the physiological state (Brown et al., 2003), acute neurological deficits like, cerebral stroke (Kokaia et al., 2006) and neurotoxic lesions (Winner et al., 2006) have been shown to trigger the multiplication of a subset of NSC progenies in the SVZ and the migration of these cells towards the incapacitated brain regions. Thus, enormous attempts have been made to characterize the regulation of neurogenesis and migration of neuroblasts in the forebrain of adult subjects as it represents a possible self-regenerative mechanism of the neurodegenerative conditions including HD (Curtis et al., 2003; Kokaia et al., 2006; Kohl et al., 2007; Kandasamy et al., 2010; Ernst et al., 2014). Here, several preclinical models of HD have been generated to investigate the roles of mutant HD gene in HD pathogenesis. The regulation of neurogenesis in NSC niches has been evaluated in R6/1 (Lazic et al., 2006), R6/2 (Kohl et al., 2007), N171-82Q (Duan et al., 2008), YAC128 (Simpson et al., 2011) and TgHD (Kandasamy et al., 2010)—genetic rodent models and in the quinolinic acid injection-induced acute rat model of HD (Tattersfield et al., 2004). Besides, adult neurogenesis has also been characterized in post-mortem brains of human HD subjects (Curtis et al., 2003; Low et al., 2011; Ernst et al., 2014). Notably, the proliferative potential of NSCs is reduced specifically in the hippocampus in most genetic models of HD (Lazic et al., 2006; Kohl et al., 2007; Kandasamy et al., 2010; Simpson et al., 2011), but no changes in the hippocampal NSC proliferation were observed in the post-mortem tissue of HD patients (Low et al., 2011). While the NSC proliferation rate was reduced in the hippocampus, the overall cell proliferation was unaltered in the SVZ of R6/1(Lazic et al., 2006), R6/2 (Kohl et al., 2007), YAC-128 (Simpson et al., 2011) mouse models and of early stage tgHD rats when compared to that of respective control animals (Kandasamy et al., 2010). In contrast, cell proliferation was found to be reduced in the SVZ of late stage tgHD rats (Kandasamy et al., 2015). Moreover, the reduced NSC proliferative capacity in the SVZ appeared to be compensated by the enhanced mitotic events of neuroblasts in late stage transgenic HD rats (Kandasamy et al., 2015). This might be also the case in the SVZ of other genetic models of HD with different grades of behavioral and neuropathological symptoms (Kandasamy et al., 2011; Velusamy et al., 2017). As a result, a vigorous migratory pattern of neuroblasts, instigated towards the degenerated striatum was highly pronounced at the expense of olfactory neurogenesis in most genetic models, which in part, mimicked the reactive neurogenesis reported in the SVZ or SEL-striatal regions in the brains of the toxic QA-injected experimental rat model (Tattersfield et al., 2004) and human HD brains (Curtis et al., 2007; Ernst et al., 2014), respectively. Taken together, reactive neurogenesis resulting from the striatal migratory event of neuroblasts seems to be a unique cellular trait, signifying the emergence of regenerative foci in the striatum of HD brains throughout the animal kingdom including humans. This abnormal proliferation of neuroblasts in the SVZ and their migration into the vulnerable striatum have recently been recognized as “reactive neuroblastosis” in the tgHD rat model (Kandasamy et al., 2015; Velusamy et al., 2017). Apparently, in this context, the process of neurogenesis is prematurely terminated, i.e., the cell die before they mature and before they integrate into the striatal circuitry (Figure 1). In the human HD brain, the situation seems to be similar (Ernst et al., 2014). Interestingly, the phenomenon of reactive neuroblastosis seems not to be restricted to HD pathology, but has recently been reported also in brains of ALS patients associated with dementia (Galán et al., 2017). Taken together, the proposed reactive neuroblastosis event observed in the striatum of the adult brain requires a great scientific consideration as it provides a fresh perspective on neurobiology of aging and disease, epitomizing a potential therapeutic target for *in vivo* forebrain regeneration. Hence, the functional relevance of reactive neuroblastosis and its consequence should be carefully considered in progressive neurodegenerative disorders. Likewise, where appropriate, the reactive neuroblasotosis process needs to be investigated in acute neurological complications such as stroke, seizure, neuroinflommatory disorders and traumatic brain injuries.

## TGF Beta Signaling and Huntingtin Protein as Potential Mediator of Cellular Events

As mentioned above, SVZ-striatal neurogenesis in HD is characeterized by reduced stem cell activity, reactive neruoblastosis and by premature death of the young neurons. An essential question is of course the physiological and molecular regulation of these events. We are postulating a framework that integrates physical activity, transforming growth factor-beta1 and mutant huntigtin protein as potential regulators. Physical exercise has been unequivocally shown to prevent cognitive decline by facilitating neurogenesis specifically in the hippocampus of healthy adult brains (van Praag et al., 1999). However, physical exercise has failed to ameliorate impaired hippocampal neurogenesis in the R6/2 (Kohl et al., 2007) and N171-82Q (Potter et al., 2010) models of HD. Also, physical exercise failed to influence the SVZ derived OB neurogenesis in the healthy brain (Brown et al., 2003). Therefore, hippocampal and SVZ/OB neurogenesis are differentially affected by regulatory signaling mechanisms, and physical activity is not counteracting the impaired hippocampal neurogenesis observed in HD animal models. Interestingly, a routine physical exercise practice has accelerated pathogenesis in a marathon runner who had been diagnosed with pre-symptomatic HD (Kosinski et al., 2007). The reason for this is unclear, but signaling mediated by TGF-beta might be crucially involved. First, physical exercise has been shown to induce the expression of TGF-beta in the normal healthy brain and to suppress spontaneous motor activity (Inoue et al., 1999). Second, physiological levels of TGF-beta and its downstream signaling pathway have been linked to the regulation of NSC’s self-renewal, migration, integration and survival of neuroblasts in the normal adult brain (Kandasamy et al., 2014), and experimentally elevated levels of TGF beta in the adult brains hindered the proliferative potential of NSCs and neurogenesis in the hippocampus (Buckwalter et al., 2006; Wachs et al., 2006; Aigner and Bogdahn, 2008). Similarly, analysis of phosphorylation events of Smad2, a downstream component of TGF-beta signaling in the hippocampal stem cell niches of R6/2 mice and tgHD rats, revealed that elevated levels of TGF beta/Smad2 signaling play a crucial role in the induction of quiescence of NSCs leading to reduced hippocampal neurogenesis (Kandasamy et al., 2010). Third, an increased Smad2 phosphorylation observed in the ectopically migrating neuroblasts from the SVZ towards the striatum of HD brain indicated a possible role of TGF-beta signaling in the migration/early differentiation of neuroblasts (Kandasamy et al., 2015; Figure 1). In the healthy brain, this might well promote structural and functional differentiation and maturation of neurons, however, in the HD brain, this might be completely different: although very speculative, involuntary hyperkinetic movements (Chorea) might cause increased levels of TGF-beta in the HD brains. In consequence, as Bowles et al. (2017) had shown, this might lead to the upregulation of mutant huntingtin protein through the activation of Smad3, a binding partner of Smad2, and the increased mutant huntingtin protein levels might trigger the apoptotic events in SVZ derived neuroblasts or young immature neurons (de Luca et al., 1996; Schuster and Krieglstein, 2002). In summary, the elevated levels of TGF-beta in the HD brain might on one hand cause a lower level of NSC activity, and through elevation of mutant hintingtin expression cause a premature death of differentiating neuroblasts regardless of the origin of the neuroblasts. For example (Magnusson et al., 2014) demonstrated that a subset of astrocytes have the capacity to produce new neurons in the striatum independent of the SVZ, while a striatal specific stem cell niche is yet to be recognized (Magnusson et al., 2014). Also here, elevated TGF-beta levels might finally reduce the levels of neuronal production and the survival of the new neurons. Taken together, it can be proposed that therapeutic physical exercise and/or hyperkinetic movements in HD subjects might play a major role in impeding adult neurogenesis through elevated TGF-beta/Smad2 signaling, which might elevate the expression of mutant huntingtin protein in neuroblasts leading to premature death of these cells. In contrast, in the healthy brain, physical exercise induced TGF-beta/Smad signaling may act in synergy with huntingtin protein, and this in turn can lead to terminal differentiation of neuroblasts resulting in functional neurogenesis. The source of the elevated TGF-beta levels upon physical exercise in the healthy brain is not known, under pathological situations, activated microglia and reactive astrocytes are potential sources of TGF-beta (Lindholm et al., 1992; Doyle et al., 2010; Kandasamy et al., 2010).

## Immunological and Non-Neurogenic Roles of Reactive Neuroblastosis in the Adult Striatum

Reactive neuroblasts in the HD brains might be immunologically active and modulate microglia activities. Microglia have been strongly implicated in immune surveillance and synaptic pruning (Kettenmann et al., 2013), and they are responsible for synaptic integration of new-born neurons, thereby supporting the neuroplasticity of adult neurogenesis (Ekdahl, 2012). A substantial number of reports suggested that disruption of microglial functions along aging and neurodegenerative processes leads to synaptic disruptions, neuronal loss and, consequently, to cognitive impairments (Morris et al., 2013). The activated microglia mediated prolonged neuroinflammatory responses have been well recognized in HD (Sapp et al., 2001; Crotti et al., 2014).

In an independent attempt to characterize microglial cells in the HD brain using ionized calcium binding adaptor molecule 1 (IBA 1) staining, the SVZ-region of early stage tgHD rats showed an indication for microglial activation compared to controls. In contrast, a drastic reduction in the number of microglial cells was observed in parallel with the invasion of neuroblasts in the striatum of late stage tgHD rats compared to the early stage and that of age matched controls (unpublished own data). One possible reason might be a non-cell autonomous mechanism, by which the glial specific expression of mutant huntingtin protein can induce prolonged reactive astrocytosis and activated microgliosis (Bradford et al., 2010; Ehrlich, 2012). Abnormal glial cell activity may result in depletion of microglia and astrocytes due to phagocytosis in the pathogenic HD brains. Thus, the observed reactive neuroblasts in the SVZ deviating towards the striatum may also be an immunological response in order to compensate for the reduction in glial cells, particularly the depletion of microglia in late stage of HD.

Previously, Kohl et al. (2010), demonstrated that the expression of the mutant huntingtin protein was specifically found in the dopamine- and cAMP-regulated neuronal phosphoprotein (DARPP)-32 positive cells of the inner striatum of R6-2 mice. However, the expression of the mutant huntingtin protein appears to be delayed in the proliferating precursor cells of the striatum adjacent to the SVZ in R6/2 animals (Kohl et al., 2010). This observation closely overlaps and corresponds to the process of reactive neuroblastosis, in which, the absence of mutant huntingtin protein and pSmad2 in the neuroblasts has been proposed for the delayed terminal differentiation of new neurons in the adjacent striatum of HD subjects (Kandasamy et al., 2015).

Besides an immunomodulatory role, a concomitant experimental evidence using transcriptome analysis of NSCs suggested that induced levels of TGF beta can suppress the expression of Myelin basic protein (MBP), a marker of oligodendrocytes (Kandasamy et al., 2014). While conditional expression of the mutant huntingtin protein has been shown to induce apoptosis in oligodendrocytes, demylination can be expected in the striatum of HD subjects (Huang et al., 2015). Thus, impaired myelination process in the striatum, in part, might be responsible for the failure in the synaptic plasticity of newly generated neurons (Alizadeh et al., 2015; Bourbon-Teles et al., 2017). Taken together, it can be postulated that the selective proliferation of neuroblasts responsible for reactive neuroblastosis may partly take over neuroinflammatory functions of glial cells in the absence or dysfunction of microglia or astrocytes in the HD striatum. Besides, depletion or dysregulation of neurotransmitter inputs have been shown to induce the migration of neuroblasts towards the striatum by compromising the cell proliferation in the SVZ (Winner et al., 2006). Thus, the migrating neuroblasts might also provide an alternate source of neurotransmitters, trophic and growth factors involved in synaptic plasticity to support the function of the striatum. It certainly demands further experiments to investigate the additional roles of neuroblastosis with respects to immune defense, trophic support and synaptic pruning compared to glial cells in the adult brain. Thereby, the extra-neurogenic roles of normal and reactive neuroblasts can functionally be addressed for many acute forms of neurological diseases such as stroke and epileptic seizure.

## The Functional Roles of Neurobasts in the Striatum of the Adult Brain

A significant scientific progress has been made in understanding the physiological roles and regulation of adult neurogenesis in aging and disease (Deng et al., 2010; Couillard-Despres et al., 2011; Marschallinger et al., 2015). The generation and functional integration of the new born neurons in the adult brain enticed by physical activity (van Praag et al., 1999; Vivar et al., 2013) and environmental stimuli (Kempermann et al., 1997; Zhao et al., 2008; Ming and Song, 2011) not only contribute to neural plasticity but also facilitate brain regeneration and functional recovery upon acute brain injuries and progressive neurodegenerative conditions (Nakaguchi et al., 2011). The primary roles of hippocampal neurogenesis have been demonstrated to be linked with pattern separation, mood regulation, contextual learning and memory processes (Clelland et al., 2009), whereas the SVZ derived neurogenesis in the OB has been implicated in odor discrimination and sexual desire (Sakamoto et al., 2011; Feierstein, 2012; Hill et al., 2015). Interestingly, evidence for the occurrence of adult neurogenesis has also been established in amygdala (Jhaveri et al., 2017), hypothalamus (Paul et al., 2017) and cortex (Gould et al., 1999) responsible for fear memory, HPA-axis and motor control, respectively. Thus, different brain regions sustain the regenerative potential to establish new neurons in the adult stage.

The striatum is a central part of the basal ganglia, responsible for the functionality of limbic system attributed to voluntary motor control, reward process, cognitive functions and behavior (Graybiel and Grafton, 2015; Yager et al., 2015). It integrates inputs from the cortex, thalamus, substantia nigra and brain stem and processes them to the relevant functional regions of the brain, including hippocampus and OB, to regulate diverse neurocognitive and motor functions (Martinez-Marcos et al., 2005; Ross et al., 2011; Calabresi et al., 2014; Vicente et al., 2016). While roles for the striatum in motor function have been well established (Lenz and Lobo, 2013; Hutton et al., 2017), its functional role on cognition including learning and memory synchronized together with hippocampus and OB remain unclear (Setlow et al., 2003; Albouy et al., 2013). The generation of neuroblasts in the striatum might be responsible for integrating and processing these external stimuli. For example, physical exercise (van Praag et al., 1999) and sex pheromones (Hoffman et al., 2015) have been shown to induce hippocampal neurogenesis under healthy condition. While defects in the OB neurogenesis and olfactory dysfunctions have been reported in the HD (Nordin et al., 1995; Moberg and Doty, 1997; Hamilton et al., 1999), the regulation of hippocampal neurogenesis mediated by pheromones through the olfactory route may not be possible. Moreover, physical exercise is also not beneficial in promoting the hippocampal plasticity under HD condition (Kohl et al., 2007) thereby suggesting a possible disconnection of the hippocampus and OB from the sensorimotor processes of the striatum in HD. Likewise, the presence of neuroblasts in the striatum has been characterized in the normal human brain, but the striatum of human HD brain has been found to be devoid of neuroblasts (Ernst et al., 2014). While the DCX positive cells have been shown to possess electrophysiological properties and secrete trophic factors (Liu et al., 2009; Couillard-Despres et al., 2011; Klempin et al., 2011), striatal neuroblasts that are positive for DCX may play a major role in transmitting the physical exercise and pheromone-mediated sensorimotor signals to the hippocampus of the normal adult brain. Thereby, the striatum might act as a cellular hinge or tissue juncture of many peripheral and environmental inputs and process it to other brain region via neuroblasts. It has been well established that the hippocampus is functionally connected to the striatum (Albouy et al., 2013). With respect to the physical activity mediated external stimuli, the regulation of hippocampal neurogenesis might be facilitated by striatal neuroblasts through a possible limbic-motor interface. Thus, gradual decline in physical activities and deficits in environmental stimuli along with the reduced cerebral metabolic rate and aging systemic milieu may partially explain a possible reason for failure in survival of neuroblasts in the striatum of normal aging human brains. The other newly identified adult neurogenic regions such as cortex, amygdala and hypothalamus have classically been known for their connections with the striatum (Macpherson et al., 2014). Thus, the striatal neurogenesis may also be collectively associated with motor memory, fear memory, olfactory memory, contextual memory, hormonal regulation of memory. Taken together, the neuroblasts in the striatum may represent a central axis for a sensorimotor pathway to regulate various neurolplastic functions of the brain including cognition.

## Possible Limiting Factors of the Analysis of Neuroblasts in the Adult Brain

Obviously, there are still limitations in the techniques that are used to detect and to analyze neurogenesis. Recently, Jonas Frisén and colleagues have implemented the radioactive carbon dating procedure to estimate the persistence of neurogenic process in the striatum and RMS-OB path along the aging process and in HD human brains (Bergmann et al., 2012; Ernst et al., 2014). Though there was no traceable amount of neuroblasts observed in the RMS, turnover of a interneuronal population in the striatum was evident in adult human brains (Ernst et al., 2014). The striatal turnover of neuroblasts is likely to be originated in the SVZ, but the survival of neuroblasts was found to be diminished in the striatum of both the healthy and HD human subjects (Ernst et al., 2014). Eventually, the disoriented neuroblasts originated from the SVZ failed to differentiate, integrate and survive in the human striatum (Ernst et al., 2014), confirming the previous reports on R6/2 mouse (Kohl et al., 2010) and tgHD rat -models of HD (Kandasamy et al., 2015). Interestingly, validation of neurogenesis in the RMS-OB path using radioactive ^14^C dating enforced the view that the human OB is devoid of ongoing neurogenesis in the adulthood (Wang et al., 2011; Bergmann et al., 2012). This is somewhat contradictory to a previous report in which (Curtis et al., 2007) using BrdU labeling method demonstrated that the migration of neuroblasts through the RMS contributes to neurogenesis in the OB of the human brain. Both paradigms for tracing newly divided cells in the human brain, either using nucleotide analogs or radioactive ^14^C, have their own merits and limitations. For example, it has generally been believed that intake of food represents the primary source of carbon in the human body. However, the olfactory epithelium of the nasal mucosa is connected with the OB through the filia olfactoria, in which a fraction of atmospheric air and volatile pheromones are able to reach the OB during respiration (Coates, 2001; Lahiri and Forster, 2003; Sun et al., 2009; Gao et al., 2010). As reported earlier, the OB can sense the atmospheric CO_2_ (Hu et al., 2007; Gao et al., 2010; Carlson et al., 2013) where it can diffuse into bio-available metabolites in the brain through a CO_2_ fixation process (Berl et al., 1962; Pincus, 1969; Lahiri and Forster, 2003; Scott, 2011). Thus, a considerable amount of radioactive ^14^C depletion can be expected specifically in the genome of mitotically active cells in the human OB due to the well-known “Suess effect” (Stenström et al., 2010; Graven, 2015; Lång et al., 2016), through which radioactive ^14^C can be exchanged by normal ^12^C from the atmospheric CO_2_ and some organic volatile compounds such as pheromones (Pinto, 2011; Cazakoff et al., 2014; Ajmani et al., 2016). Moreover, the radioactive ^14^C decay has been considered a spontaneous and highly random process, as different ^14^C atoms can radically be reverted into ^14^N atoms at different degrees due to a subatomic transmutation process. The safety guideline and dosimetry of radioactive elements suggest that ^14^C is a low energy beta emitter and therefore a short-term external exposure may be harmless (Pauling, 1958; Kim et al., 2010). However, according to Pauling, it would also be expected that a prolonged cell intrinsic emission of ^14^C radiation and accumulation of ^14^C incorporated cells in close proximity might intensify the dose of radiation (Pauling, 1958). In turn, a magnitude of radiation discharged from a large quantity of ^14^C atoms has been suggested to induce irreversible mutational events in genome, leading to carcinogenesis or apoptosis in humans (Pauling, 1958). Hence, radioactive ^14^C based tracing of neurogenesis in the human brain may require further validation to exclude, if present, any false negative results or artifacts. Similarly, the incorporation of halogenated thymidine analogs by apoptotic cells (Cooper-Kuhn and Kuhn, 2002), their toxic effects on cell viability (Lehner et al., 2011), DNA instability or repair mechanisms and phagocytosis events rendered by microglia also require a careful consideration (Rakic, 2002). Nevertheless, it has widely been accepted that neurogenesis occurs in adult brain as it provides a foundation for neural plasticity across the animal kingdom (Ming and Song, 2011; Velusamy et al., 2017). While multiple intrinsic and extrinsic stimuli have been shown to regulate adult neurogenesis in the hippocampus and the SVZ-OB, its regulation in the striatum has become an important topic of elucidation.

## Conclusion

The naturally occurring stem cell mediated neuroregenerative processes in the adult brain under physiological condition provides a clue in ascertaining a potential therapeutic target for many neurodegenerative disorders including HD. The occurrence of SVZ derived neuroblasts as a part of neurogenic event in the striatum of both animal and human brains, signifies an impetuous self-restorative attempt of the adult brain. A growing body of evidence supports the idea that the SVZ derived neuroblasts in the striatum may provide structural and neurophysiological alternative for trophic support and neuronal loss and thereby it may contribute in restoring the motor and cognitive functions that are lost in HD. Therefore, investigation into the molecular mechanism involved in reactive neuroblastosis at the levels of cell proliferation, migration, integration and survival in the damaged area may provide a clue for therapeutic intervention not only for treating HD but also for a variety of neurological deficits such as stroke, Parkinson’s disease and Alzheimer’s disease.

## Author Contributions

MK: manuscript preparation and writing, preparation of figure. LA: manuscript preparation and writing.

## Conflict of Interest Statement

The authors declare that the research was conducted in the absence of any commercial or financial relationships that could be construed as a potential conflict of interest.
